# 2526. *Ex Vivo* Assessment of Sulbactam-Durlobactam (SUL-DUR) Clearance during Continuous Renal Replacement Therapy (CRRT)

**DOI:** 10.1093/ofid/ofad500.2144

**Published:** 2023-11-27

**Authors:** Yasmeen Abouelhassan, Yuwei Shen, April Chen, Xiaoyi Ye, David P Nicolau, Joseph L Kuti

**Affiliations:** Hartford Hospital, Hartford, Connecticut; Hartford Hospital, Hartford, Connecticut; Entasis Therapeutics, Waltham, Massachusetts; Hartford Hospital, Hartford, Connecticut; Hartford Hospital, Hartford, Connecticut; Hartford Hospital, Hartford, Connecticut

## Abstract

**Background:**

Optimal dosing of antibiotics in patients on CRRT is complicated by factors that can influence drug adsorption and clearance including filter type, CRRT mode, effluent rate (ER), and the drug’s properties itself. SUL-DUR is a novel combination antibiotic under development for management of *Acinetobacter baumannii* infections. We sought to characterize SUL and DUR adsorption and transmembrane clearance (CL_TM_) in an *ex vivo* CRRT model.

**Methods:**

CL_TM_ was determined in hemofiltration (CVVH) and hemodialysis (CVVHD) modes using the Prismaflex M100 and HF1400 hemofilter sets. One liter of heparinized bovine blood was allowed to circulate in the Prismaflex CRRT system for 10 minutes. SUL and DUR were then added to achieve a plasma concentration of 30 mg/L, the average maximum concentration achieved in humans following SUL-DUR 1g/1g q6h as a 3h infusion. Pre-filter blood, post-filter blood and effluent samples were collected at 0, 10, 30, and 60 minutes to determine SUL and DUR concentrations. Combinations of filters, modes, and replacement fluid were tested in triplicate at effluent rates of 1, 2 and 3 L/h. The sieving coefficient (SC) for CVVH and the saturation coefficient (SA) for CVVHD were calculated to determine CL_TM_. Adsorption was measured through a closed loop system bypassing effluent elimination. Multiple linear regression was used to determine SUL and DUR CL_TM_ as a function of ER, filter, and mode.

**Results:**

SUL and DUR adsorption was minimal at 10% for both drugs. Mean ± standard deviation initial SUL and DUR concentrations were 28.6 ± 2.5 and 27.4 ± 1.9 mg/L, respectively. The overall mean SC/SA across different modes, filters, and ERs was 1.0 and 0.9 for SUL and DUR, respectively. Multiple linear regression demonstrated that the ER was the primary driver (p< 0.001) of CL_TM_ for both drugs based on the equations: SUL CL_TM_ (L/h) = -0.0123 + (1 x ER), R^2^ = 0.998; DUR CL_TM_ (L/h) = 0.0346 + (0.91 x ER), R^2^ = 0.958.

**Conclusion:**

SUL and DUR were efficiently cleared by both CVVH and CVVHD through M100 and HF1400 filters. The clearance of both drugs during CRRT was dependent primarily on the ER. When incorporated into established population pharmacokinetic models, these data can be used to estimate CL_TM_ and devise dosing recommendations for SUL-DUR for patients requiring CRRT.

**Disclosures:**

**Xiaoyi Ye, MD**, Sanofi Genzyme: Honoraria **David P. Nicolau, PharmD**, Allergan: Advisor/Consultant|Allergan: Grant/Research Support|Cepheid: Advisor/Consultant|Cepheid: Grant/Research Support|Merck: Advisor/Consultant|Merck: Grant/Research Support|Pfizer: Advisor/Consultant|Pfizer: Grant/Research Support|Shionogi: Advisor/Consultant|Shionogi: Grant/Research Support|Tetraphase: Advisor/Consultant|Tetraphase: Grant/Research Support|Venatorx: Advisor/Consultant|Venatorx: Grant/Research Support|Wockhardt: Advisor/Consultant|Wockhardt: Grant/Research Support **Joseph L. Kuti, PharmD**, bioMeriuex Inc.: Grant/Research Support|Entasis Therapeutics: Grant/Research Support|Merck & Co, Inc: Grant/Research Support|Shionogi Inc: Advisor/Consultant|Shionogi Inc: Grant/Research Support|Shionogi Inc: Honoraria

